# Aortic Replacement for a Crawford II Thoracoabdominal Aneurysm With a Right-Sided Descending Aorta: A Case Report

**DOI:** 10.7759/cureus.68991

**Published:** 2024-09-09

**Authors:** Yuki Takagi, Toshihito Gomibuchi, Noburo Ohashi, Yuko Wada, Tatsuichiro Seto

**Affiliations:** 1 Division of Cardiovascular Surgery, Shinshu University Hospital, Matsumoto, JPN; 2 Department of Surgery, Division of Cardiovascular Surgery, Shinshu University Hospital, Matsumoto, JPN

**Keywords:** dissecting aortic anerysm, marfan's syndrome, excluded aneurysm, right-sided descending aorta, thoracoabdominal aortic aneurysm

## Abstract

A 53-year-old woman was diagnosed with a Crawford II thoracoabdominal aortic aneurysm involving the right-sided descending aorta. The patient underwent aortic replacement via a thoracoabdominal approach. The right-sided descending thoracic aortic aneurysm was excluded. The patient had a favorable postoperative course. The excluded thoracic aneurysm had completely thrombosed without intercostal inflow. The right-sided descending aorta is a rare malformation. The exclusion technique was appropriate because there was no retrograde flow from the intercostal arteries and the Adamkiewicz artery originated from the lumbar region.

## Introduction

Thoracoabdominal aortic aneurysms (TAAAs) present a significant surgical challenge, particularly when compounded by rare anatomical variations or pre-existing medical conditions. Among these challenges, the presence of a right-sided descending aorta is an unusual anatomical anomaly that complicates both diagnosis and surgical intervention [1]. Traditionally, the surgical approach to TAAAs involves open surgical techniques, such as thoracoabdominal aortic replacement. A recent advance in endovascular repair methods is thoracic endovascular aortic repair (TEVAR). However, in the repair of aortic aneurysms associated with Marfan syndrome, open surgery is often preferred, taking into account the patient's overall health and the extent of the disease. Particularly when there are complex anatomical elements such as branch arteries involved, open surgery is considered more appropriate. This case report details the surgical management of a 53-year-old woman with a Crawford type II TAAA involving a right-sided descending aorta. The patient, who has a history of Marfan syndrome and multiple previous aortic surgeries, presents a complex clinical scenario. We discuss the surgical approach undertaken, the anatomical considerations, and the outcomes of this rare case. This report aims to contribute to the understanding of surgical strategies for managing TAAAs with unusual anatomical features and provide insights into potential long-term management and follow-up strategies.

## Case presentation

**Figure 1:**
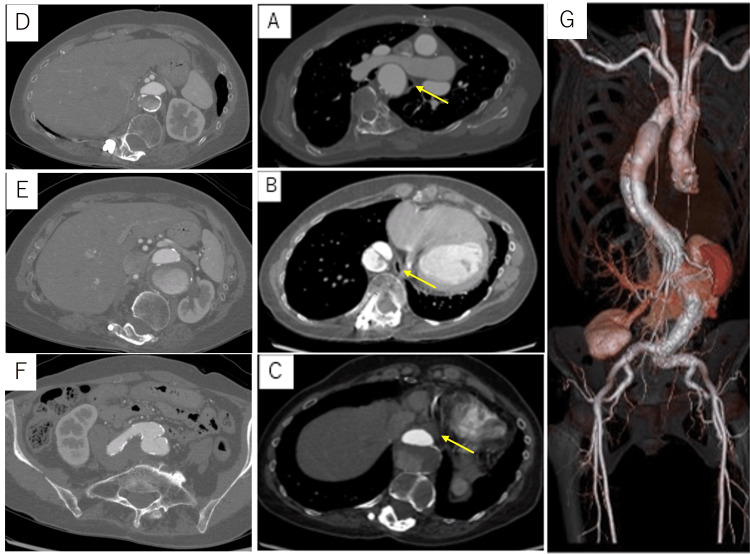
Patient’s postoperative CT findings The tortuous descending aorta was located just behind the esophagus at the second thoracic vertebrae level (A), which ran along the right side of the spinal column to the first lumbar vertebra (B), and then returned to the usual position (C). Abdominal vessels bifurcated from the true lumen (D). Dilatation of the aorta was observed from the second thoracic vertebral level with a maximum diameter of 55 mm at the ninth vertebral level (E). False lumen continues to bilateral common iliac bifurcation (F). (G) Three-dimensional CT image of the thoracoabdominal aneurysm. The arrows indicate the location of the esophagus.

A 53-year-old woman was referred to our hospital for progressive dilatation of a dissecting TAAA, which was being followed by a local physician. The patient was diagnosed with Marfan syndrome and had a history of an m-Bentall operation and total arch replacement for acute type A aortic dissection, acute type B aortic dissection, posterior spinal fusion surgery for scoliosis, and total hysterectomy for uterine fibroids. She was 159.5 cm in height and weighed 51.2 kg. The patient had unusually long fingers and toes. Computed tomography (CT) showed a tortuous descending aorta just anterior to the esophagus at the level of the second thoracic vertebra, running along the right side of the spine to the first lumbar vertebra, and then returning to its normal position. Dilatation of the aorta was observed from the second thoracic vertebral level with a maximum diameter of 55 mm at the ninth vertebral level. An entry was found in the descending aorta, and the celiac artery (CA), superior mesenteric artery (SMA), and both renal arteries (RAs) emerged from the true lumen. The Adamkiewicz artery was identified as the left second lumbar artery, originating from the true lumen (Figure 1).

**Figure 2:**
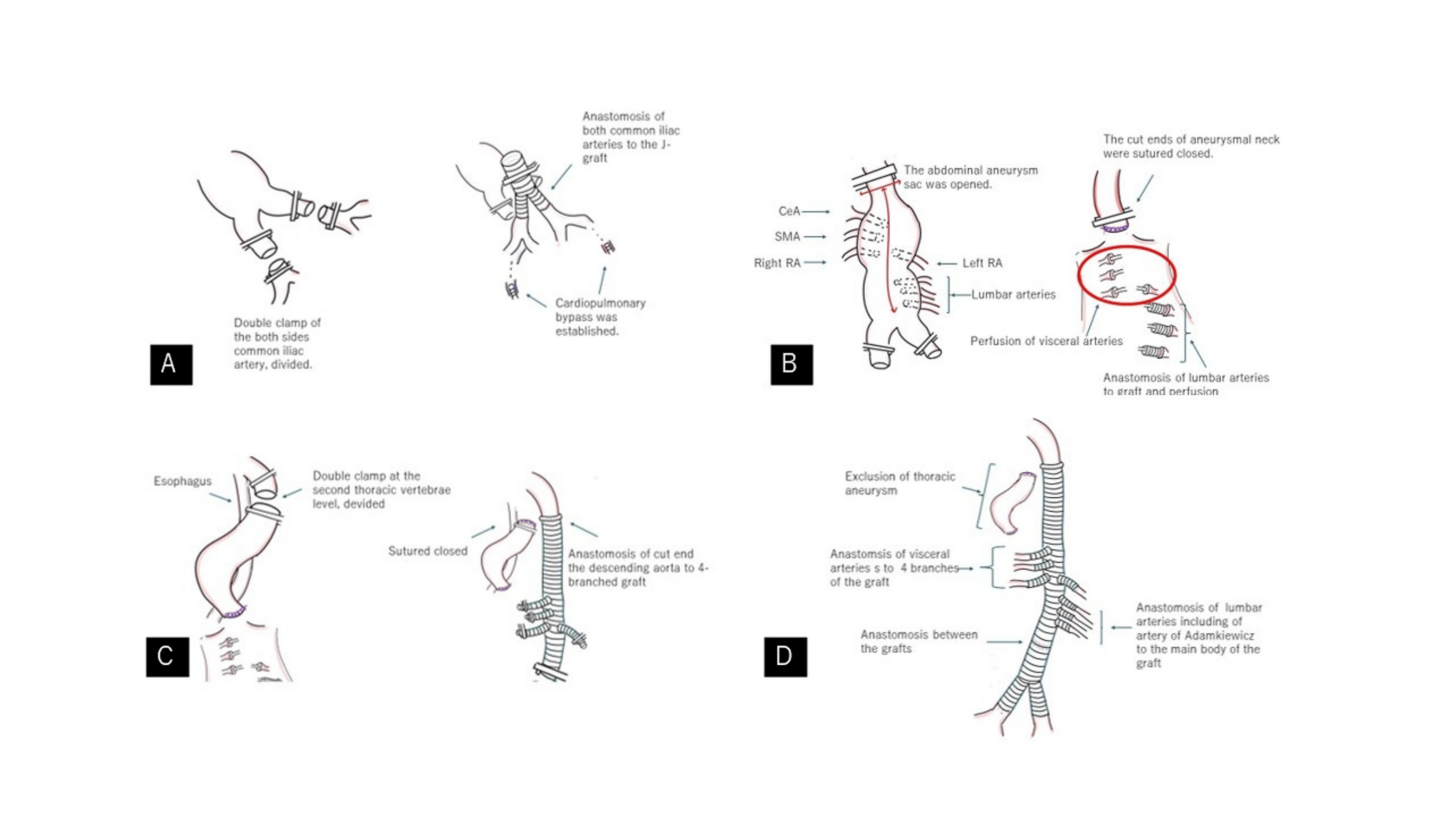
Surgical findings This figure is the original work of the authors. The bilateral common iliac arteries were double-clamped, divided, and anastomosed to a Y-shaped Dacron graft. Cardiopulmonary bypass through left femoral artery perfusion and right femoral vein drainage were established (A). The abdominal descending aorta was clamped and the aneurysm sac was opened. The celiac artery (CA), superior mesenteric artery (SMA), and renal arteries (RAs) were perfused. The first to third lumbar arteries were divided and anastomosed to the graft and perfusion was initiated. The proximal cut ends of the aneurysmal neck were sutured and closed (B). The descending thoracic aorta was double-clamped at the second thoracic vertebral level and divided. The proximal cut end was anastomosed to the graft, the distal cut end was sutured closed, and the right descending thoracic aortic aneurysm was excluded (C). Following graft anastomosis, the CA, SMA, and both RAs were anastomosed to the main body of the graft. The L1–L3 arteries were anastomosed to the main body using a graft (D).

We inserted a lumbar cerebrospinal fluid drain the day before surgery (Video 1). The patient was placed in the right-lateral decubitus position. A thoracoabdominal incision was made in the seventh intercostal space, and a retroperitoneal approach was used. Following systemic heparinization, the bilateral common iliac arteries were double-clamped, divided, and anastomosed using a Y-shaped Dacron graft (J graft 20 × 10 mm, Japan Lifeline Inc., Tokyo, Japan). Cardiopulmonary bypass through left femoral artery perfusion and right femoral vein drainage was established, and perfusion was initiated (Figure 2A). The abdominal descending aorta was clamped proximal to the CA, and the aneurysm sac was opened. The CA, SMA, and RAs were perfused. The first, second, and third lumbar arteries, including the Adamkiewicz artery, were divided and anastomosed to a Dacron graft (J graft, 11 mm; Japan Lifeline Inc., Tokyo, Japan), and perfusion was initiated. The proximal end of the aneurysmal neck was sutured and closed (Figure 2B). The descending aorta was double-clamped and divided at the level of the second thoracic vertebra. The proximal cut end was anastomosed to a four-branched Dacron graft (J graft 20 × 9 × 8 × 8 mm; Japan Lifeline Inc., Tokyo, Japan), and the distal cut end was sutured closed, excluding the right-sided descending thoracic aortic aneurysm (Figure 2C). Following graft-to-graft anastomosis, the CA-SMA and both RAs were anastomosed to the graft branches. The L1-L3 arteries were also anastomosed to the main body with an 11 mm graft (Figure 2D). Surgery was terminated after the confirmation of hemostasis. The operative time was 15 hours and 18 minutes, and the cardiopulmonary bypass time was 6 hours and 55 minutes.

**Video 1:** Intraoperative findings Findings from the establishment of the cardiopulmonary bypass until the completion of all anastomoses are shown.

**Figure 3:**
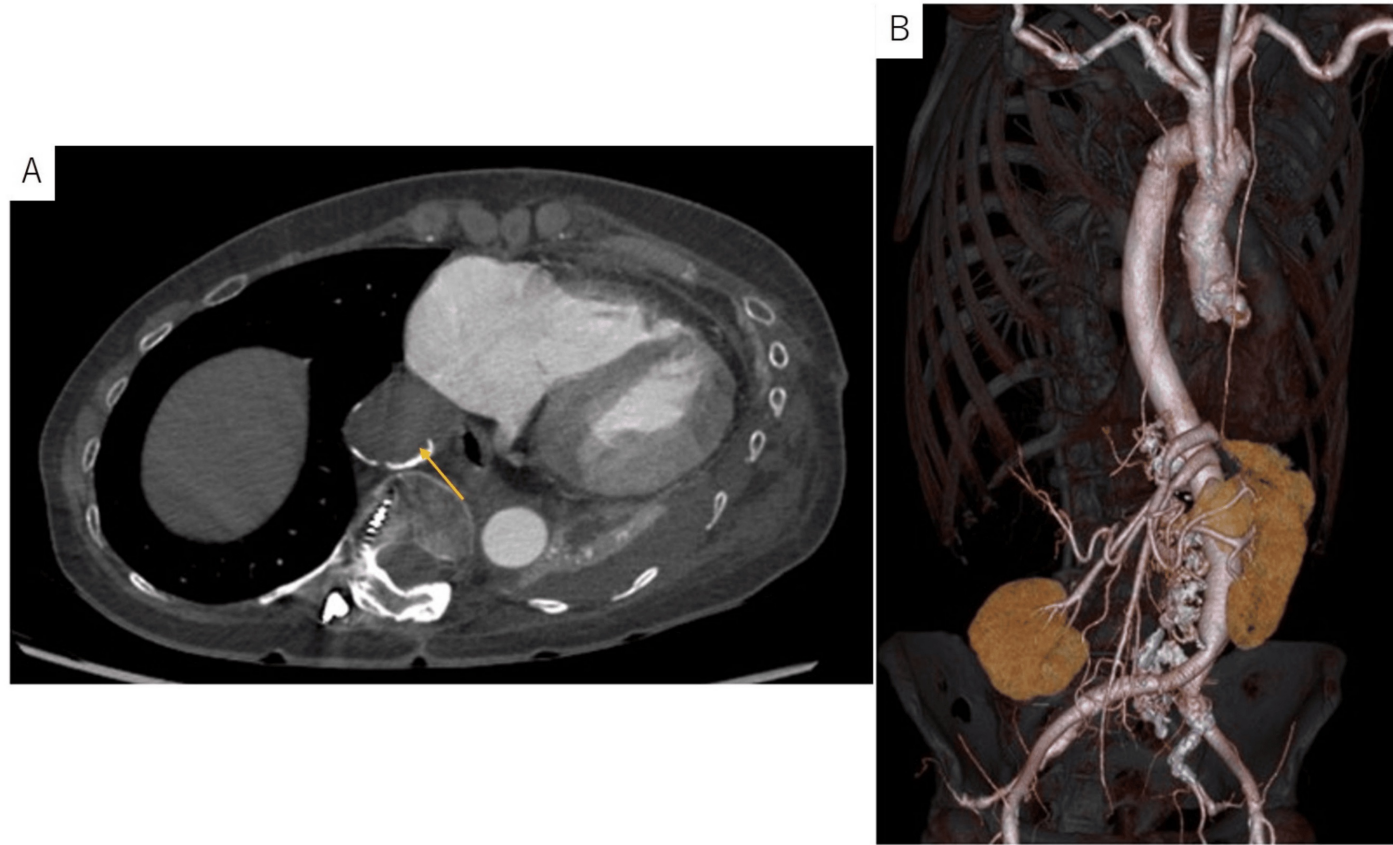
Patient’s postoperative CT findings (A) The excluded thoracic aneurysm was completely thrombosed without any intercostal inflow(arrow). (B) Three-dimensional CT image.

The patient was weaned off the ventilator on postoperative day 5 and discharged from the intensive care unit to the ward on postoperative day 7. The patient had an uneventful postoperative course and was discharged from the hospital on postoperative day 25. The excluded thoracic aneurysm was completely thrombosed with no intercostal inflow (Figure 3). 

## Discussion

A right-sided descending aorta is rare and is often associated with a right-sided aortic arch, accounting for 0.1% of all cases [1]. Of these, a right-sided descending aorta with a normal left-sided arch is even rarer and has only been reported in a limited number of cases, all of which were cases of thoracic aortic aneurysms [2-4]. It is a rare congenital malformation of the aortic arch and its branches are caused by an abnormality in the normal developmental process of degeneration, loss of a part of the aortic arch, and differentiation of the remaining part; however, there is no clear explanation for its development [1]. In the present case, the descending aorta was tortuous because of scoliosis caused by Marfan syndrome [5].

Until the 1900s, most patients underwent right thoracotomy for right-sided descending aortic aneurysms [2]. However, in recent years, with the development of stent grafting, TEVAR has been performed in an increasing number of cases [3]. Yet, there are very few surgical reports of TAAAs involving the right descending aorta. We identified a surgical case of Crawford type V by Yumoto et al. [6]. In this case, the patient underwent hybrid visceral debranching and endovascular repair in two stages. Similar to the present case, there have been no previous reports of thoracoabdominal aortic replacement in patients with Crawford type II. The patient underwent dissection from the aortic arch to the bilateral common iliac arteries and TEVAR was difficult to perform. In addition, the patient had Marfan syndrome. TEVAR is a useful treatment option for certain cases in patients with Marfan syndrome, but its applicability is often limited in cases with extensive disease or complex anatomical features considering the risk of re-dissection and Increased risk of recurrence and anatomical changes over time. This case had anatomical abnormalities and very extensive disease; therefore, we chose open thoracoabdominal replacement [7-10].

Tsutumi et al. reported a case of a right-sided descending aortic aneurysm treated with surgical exclusion; no enlargement of the excluded aneurysm was observed [4]. However, it has been reported that approximately 2.5% of patients require surgical intervention due to postoperative enlargement of the excluded aneurysm caused by reflux from the intercostal arteries [11]. An additional right thoracotomy was performed to remove the descending aortic aneurysm. In the present case, the exclusion technique was well suited because the intraoperative findings showed no retrograde flow from the intercostal arteries, and the Adamkiewicz artery was located in the lumbar region and not in the descending aorta. If the Adamkiewicz artery was located in the descending aorta, we would choose a two-stage operation for the right descending aorta with reconstruction of the Adamkiewicz artery. 

There are reports of cases in which an excluded aneurysm has become dilated 10 years after surgery, leading to reoperation [12]. Therefore, further follow-up is necessary in this case. If the excluded aneurysm becomes dilated, right thoracotomy for resection of the excluded aneurysm or embolization of the intercostal arteries with reflux should be considered.

## Conclusions

Operative graft replacement for a Crawford type II TAAA involving the right descending aorta is rare. The right-sided descending aortic aneurysm was excluded. We achieved a good postoperative course; however, further follow-up is necessary if the excluded aneurysm becomes dilated.
